# Improved quality and more attractive work by applying EBM in disability evaluations: a qualitative survey

**DOI:** 10.1186/s12909-016-0599-z

**Published:** 2016-02-29

**Authors:** Jan L. Hoving, Rob Kok, Sarah M. Ketelaar, Paul B. A. Smits, Frank J. H. van Dijk, Jos H. Verbeek

**Affiliations:** Coronel Institute of Occupational Health, Academic Medical Center, University of Amsterdam, Amsterdam, The Netherlands; Research Center for Insurance Medicine, Amsterdam, The Netherlands; Cochrane Work, Finnish Institute of Occupational Health, Kuopio, Finland

**Keywords:** Continuing medical education (MeSH) Disability evaluation (MeSH), Evidence- based medicine (MeSH), Social insurance medicine, Physicians’ practice, Occupational health (MeSH), Qualitative research (MeSH)

## Abstract

**Background:**

The uptake of evidence in practice by physicians, even if they are trained in the systematic method of evidence-based medicine (EBM), remains difficult to improve. The aim of this study was to explore perceptions and experiences of physicians doing disability evaluations regarding motivators and preconditions for the implementation of EBM in daily practice.

**Methods:**

This qualitative study was nested in a cluster randomized controlled trial (Trial registration NTR1767; 20-apr-2009) evaluating the effects of training in EBM. The 45 physicians that participated received a comprehensive 6-months training program in EBM of which the last course day included audio-recorded interviews in groups. During these interviews participating physicians discussed perceptions and experiences regarding EBM application in daily practice. In an iterative process we searched for common motivators or preconditions for the implementation of EBM.

**Results:**

Three main concepts or themes emerged after analyzing the transcriptions of the discussions: 1) improved quality of physicians’ actions, such as clients benefiting from the application of EBM; 2) improved work attractiveness of physicians; and 3) preconditions that have to be met in order to work in an evidence-based manner including professional competence, facilitating material conditions and organizational support and demands.

**Conclusions:**

Physicians trained in EBM are motivated to use EBM because they perceive it as a factor improving the quality of their work and making their work more attractive. In addition to personal investments and gains, organizational support should further facilitate the uptake of evidence in practice.

## Background

Although the use of evidence is considered good clinical practice [1], the uptake of evidence in practice by physicians, even if they have been trained in the systematic method of evidence-based medicine (EBM), remains difficult to improve [2]. This is also a problem during and after continuous medical education programs, where the final aim is to change professional behavior [3–5]. EBM – the systematic finding, appraisal and use of up-to-date research findings to support decision-making – has been advocated in several studies within the field of disability evaluation [6–8].

Recently, we showed that the use of evidence in the daily practice of physicians performing disability evaluations can be improved through the application of a clinically integrated comprehensive postgraduate EBM training program [6]. As a result of this program physicians trained in EBM used almost 10 % more evidence of sufficient quality in their disability reports compared to non-trained physicians. Likewise, knowledge and skills of EBM also showed statistically significant differences in favor of the group trained in EBM up to one year follow up.

In a similar educational EBM program among general practitioners developed by Shuval et al. [9] physicians reported that EBM enhanced the quality of their practice with regard to justifying decisions and selecting best treatment for patients. However, in another study, general practitioners in busy practices perceived using EBM resources during consultation hours as difficult and impractical [9]. General practitioners focus on diagnosis and treatment decisions, whereas physicians involved in disability evaluation mainly focus on client limitations in work or work disability resulting from a chronic disease or handicap, how these limitations will evolve and how adverse effects on work ability can be minimized [6, 7]. Given these differences in tasks, we wanted to study if the themes mentioned above are similar for physicians performing disability evaluations after participating in a EBM course [6, 8].

In contrast to other medical specialties the consequences of using evidence-based decisions do not only affect the judgments of work disability evaluations themselves but are also vital for the health, well-being, work participation and financial compensation of the client and have a substantial social and financial impact for workers. In this survey we explored experiences and perceptions regarding EBM practice in disability evaluation. The group of physicians doing disability evaluations who participated in comprehensive formal education in EBM was not only aware of the theoretical possibilities of EBM, but also had time to experience opportunities and problems in practice. For this reason, the central aim of this study was to explore the perceptions and experiences of physicians doing disability evaluations regarding motivators and preconditions for the implementation of EBM in daily practice. From the physicians’ answers we also hoped to learn on what aspects our future EBM training and implementation efforts could be improved.

## Methods

### Design and participants

This survey study was nested in a cluster randomized controlled trial (RCT) in which we evaluated a clinically integrated training program in EBM which successfully improved the use of evidence in disability evaluation in the intervention group compared to the control group without a training program [6]. In this cluster RCT approval was sought from the research ethics committee of the Academic Medical Center, University of Amsterdam. However, as this constituted the evaluation of an educational intervention with physicians, written exemption of ethics approval was received from the committee secretary. All participating physicians in this trial signed an informed consent form (Trial registration NTR1767: 20-apr-2009). They were all working for the Dutch National Institute for Employee Benefit Schemes (UWV) and performed disability evaluations as a substantial part of their job. The participants were selected by their peer group leaders and came from all different parts of the Netherlands.

In total 45 of 59 physicians who had completed the six-month EBM training program participated in this end-of-program qualitative survey in the form of group discussions. The group discussion design was considered convenient and feasible, given the non-sensitive nature of the discussion topics. The mean age of the complete intervention group was 49.7 years (sd 7.1). They had been employed for a mean of 16.4 years in their current job and only one quarter had previous experience with EBM, mostly consisting of a one-day introductory course [6].

### Group discussions

Focus group methodology was used to discuss physicians’ perceptions and experiences regarding EBM. In total, nine group discussions (average duration 1 h) and two plenary discussions (average duration 1.5 h) were conducted at the end of the EBM educational program to explore perceived motivators and preconditions. The focus groups consisted of on average five physicians and all group and plenary sessions were audio-recorded in Dutch. Experienced tutors (JH, PS, RK) supervised these nine group meetings, facilitating the process and encouraging participants to engage in the discussions but did not take part in the discussions themselves.

Using input from personal action plans (see Appendix), which were completed as home assignments by the participating physicians, the group discussion evolved around the topic of how physicians pictured themselves as being active EBM practitioners in the future, exploring the perceived motivators and preconditions to the application of EBM in their practice.

In every group, participants read out their plans, discussed experiences and perceptions, asked clarifying questions and gave feedback to each other. Additionally one group participant in each group took notes during the meetings so that findings could be introduced in the plenary discussion following the group discussions.

During two plenary discussions, one person from each group reported on the group’s findings, which allowed the members of the other groups to further question, add to, or explain perspectives and to clarify ideas. The plenary discussions allowed for alternative perceptions that had not been expressed before to be put forward.

### Data analysis

One of the researchers (SMK) transcribed the audio-recordings of the 11 discussions verbatim in Microsoft Word. The software program MaxQDA was used for data analysis. Coding of categories and themes was performed in Dutch by two researchers (SMK and RK) and checked by a third member of the research team (JLH). Themes and quotations were translated in English afterwards.

The analysis was performed in phases using the three types of coding originally proposed by Strauss and Corbin [10]. In the first phase – the open coding phase – every text fragment that could provide an answer to the research question was given a code. In the axial coding phase, relations between categories and larger concepts were sought. When necessary, new categories were coded. Finally, in the selective coding phase, themes were organized to formulate an answer to the research question. Iteratively, three main concepts or themes emerged along which perceptions, experiences and other discussion elements could be categorized. The content, descriptions, titles and final selection of the themes and subthemes were checked and discussed by the research team in each phase. We used the items of the COREQ checklist for improving the quality of reporting qualitative research [11].

## Results

Three main concepts or themes emerged after analyzing the group discussions representing both motivators and preconditions for implementation: 1) improved quality of physicians’ actions; 2) improved work attractiveness; and 3) preconditions to work in an evidence-based way including professional competence, facilitating material conditions and organizational support and demands. In Table 1 and in the following paragraphs we present the main themes and subthemes, supported by verbatim quotations.

**Table 1:** Themes and subthemes identified as perceived to contribute to continued use of EBM by physicians

1) Improved quality of physicians’ actions
• Keeping up-to-date improves • Services of the organization improve • Consultation of colleagues improves • Better able to underpin decisions with evidence • Clients will benefit from the application of EBM
2) Work attractiveness of physician improves
• Work becomes more attractive • More appreciation and job satisfaction
3) Preconditions to work in a evidence-based way
• *Professional competence:* efficacy, motivation and collaboration on EBM • *Facilitating material conditions*: access to literature, education in EBM, helpdesk, databases and tools for EBM • *Organizational support and demands*: sufficient time and managerial support; adequate quality assurance policy

1. Improved quality of physicians’ actions

The subthemes below all relate to the quality of the care by the physicians. Some quality aspects are linked to a perceived improved quality of the input according to Donabedian’s scheme [12] such as keeping physician’s knowledge up to date. Other quality aspects refer to improved process characteristics such as improved consultation with other medical disciplines, or better processing of the evaluations in favor of the organization. Again other themes are related to the output such as better advice to the client, or to the final outcome such as improvement in the health of the client, improved work ability, faster or sustainable return to work, or a lower number of disability pensions.

### Keeping up to date improves

Applying EBM in practice assisted participating physicians in keeping up with developments in the field of disability evaluations because it enabled them to search for relevant evidence to fill gaps in their knowledge in a more focused way. *“You’ve graduated in medicine but you can’t rely on that knowledge for the next 40 years”.* (inter 1, para 302) Physicians viewed EBM

*“… as something to help me keep up to date, to supplement my knowledge, and then perhaps it’s not always for the sake of underpinning a case study but so that you know just that little bit extra about a certain syndrome in terms of treatment, prognosis, or whatever. That you say, well instead of having my head buried in books, I can still come up with up-to-date information”.* (inter 5, para 451)

### Services of the organization improve

Due to better prognostic estimates by participating physicians, applying EBM in practice also increased the efficiency and effectiveness of organizations providing (social) insurance benefits such as the Dutch insurance authority. Scheduling more realistic re-examinations led to a better use of capacity and a higher degree of customer satisfaction according to the physicians.

*“That you’re able to say, right after the first assessment, well yes the chance that this will improve is zero. So if that man or woman is then assessed as being 100 % unfit to work, so be it. It doesn’t make any sense at all to continue to invite them year in, year out … and that saves time too, which is also an advantage for the organization. A better prognostication results in more efficient working”.* (inter 1, para 138–140)

### Consultation with colleagues improves

Since the participating physicians performing disability evaluations had access to the same and up-to-date knowledge (evidence), they considered themselves better able to engage in written or oral discussions with colleagues from the curative sector, such as general practitioners or medical specialists.

*“Sometimes you want to discuss things with a treating physician, for example that you believe that the therapy being used has had absolutely no effect for the past two years, and in such a case it’s extremely useful if you underpin that through EBM. This puts you in a much stronger position during the discussion”.* (inter 7, para 52)

According to the participating physicians, discussions with fellow physicians in other disciplines, but also with labor experts and reintegration counselors, was improved by making use of evidence.

*”So you do that by giving examples, which increases your power of persuasion, or you get evidence through EBM. And you can convince your colleague with it”.* (inter 8, para 379)

### Better decisions underpinned by evidence

Physicians considered it important to continue to use and improve their EBM skills since, in their opinion, this raised the quality of their work. Through the EBM methodology participating physicians felt better able to substantiate their disability assessment, which in their perception was also what would be expected of them by the most important stakeholder, the client:

*“We see evidence-based primarily as a means to an end, a means for achieving a better claim assessment. Actually, we also believe that our clients deserve that, that we perform our claim assessment in a more professional manner, be better able to underpin it through evidence”.* (inter 11, para 78)

### Clients will benefit from the application of EBM

The participating physicians found that in a number of cases, the client benefitted from the application of EBM in the disability evaluation, and led to better health care. Examples given by the physicians were that EBM enabled a more critical look at whether the correct interventions had been applied during the long period of sickness absence and whether any remaining (effective) interventions could be recommended.

*“Sometimes, when you see that recovery is stagnating, for example, then you have the tendency to talk to each other and say, hey is that really the right treatment, and you really want to try to motivate the (treating) physician to do something. And if you have powerful arguments, backed up by a good story, then maybe you can achieve more”.* (inter 7, para 58)

Occasionally, physicians felt the need to look up evidence to counterbalance any information from the internet that clients brought with them, which was thought to work in the clients’ favor:

*“Our clients are getting more and more vocal, they look things up on the Internet, but these sites are often fairly dubious. And in that case, it’s comforting to be able to say that, based on our professional expertise, and from the research that is available to us, the opposite appears to be the case, for example, or even to confirm what the client is claiming. That certainly adds significant value, for our clients too”.* (inter 11, para 178)

Participating physicians indicated that better estimations of the duration of an illness or the corresponding course of the work ability were possible if there was good prognostic evidence at hand, which could also contribute to the quality of life of a client:

*“And you get a totally different picture of certain problems, an older man with metastatic rectal cancer (visiting me) during surgery hours who was still working… (there were) more metastases … I looked that up, interesting … that even then there’s a chance of being cured when it’s recidivistic metastasis … And he really wanted to carry on working … if he’s lucky then he’ll be one of the 30 % that survives … And in that case he’ll also keep his job”.* (inter 5, para 376–399)

In addition, physicians believed communicating evidence regarding a client’s improvement in future work capacity supported patients to retain their job and stimulated a more optimistic perspective on the chances on the job market.

2. Improved work attractiveness for physicians

We identified two subthemes contributing to the work attractiveness for physicians; one being more direct and intrinsic way, and the other more indirect through the appreciation from colleagues of other disciplines or from managers.

### Work becomes more attractive

By applying EBM in practice, and make quick use of knowledge sources, the learning process of physicians was encouraged and their understanding was enriched, which resulted in their work becoming more enjoyable and wide-ranging:

*“Why do we do what we do, we want to be able to find relevant literature quickly for the questions we have. These are questions that we particularly need in the context of case treatments. But also out of personal interest, I think that if the physician is only interested in what he is served up on a daily basis, that’s not quite enough. So where there are gaps in our own personal knowledge, we have to be able to find the relevant literature quickly”.* (inter 11, para 60)

### More appreciation and job satisfaction

By applying and sharing EBM knowledge, physicians also indicated more appreciation from colleagues, clients, and possibly from their manager. Because participating physicians considered themselves skilled EBM practitioners they believed they could influence colleagues to become positive toward applying EBM in practice and thus enhance work satisfaction.

*“So if you can be a missionary for EBM, through case studies, or via health care peer review, or via … showing what you can achieve as an individual … at some point it will be picked up on a larger scale … what we call the snowball effect”.* (inter 1, para 169–172)

*“We want to be seen to be applying the EBM vision in our direct environment, for example in the case study groups, where we want to show how we are working with EBM, what you can achieve with EBM. And we want to pass on our enthusiasm to our colleagues so that they also get working with it”.* (inter 11, para 60)

3. Preconditions to work in an evidence-based way

Other subthemes related to preconditions to be fulfilled in order to be able to apply EBM, include professional competence, facilitating material conditions and organizational support and demands.

### Professional competence: efficacy, motivation and collaboration on EBM

A factor that does not encourage evidence-based working is that some physicians, even after a 5-day course, did not not feel fully competent to apply EBM in practice or thought critical assessment of the evidence was a difficult ask.

*“We haven’t even talked about the appraisal of the articles … that always gets pushed to the back of the queue, and then you don’t get around to it. Just like that article today, I really had to tell everybody at home to keep quiet, no noise, no radio, because otherwise I just can’t read it. Is that just me or…”* (inter 10, para 440–452)

Physicians observed that it was necessary to continue to apply EBM in practice so that the acquired skills and knowledge did not become outdated, preferably in groups which created more commitment: *“You have to carry on doing it … use it or lose it”.* (inter 5, para 278)

*“… in which case you could of course agree to meet up with each other once every two weeks for an hour, maybe discuss part of an assignment, and for the next meeting you do some studying beforehand, so that you’re forced to do something in the period between meetings. But you arrange to do this in agreement with each other, so there’s more of a commitment … If I don’t do that then I know for sure that it just won’t work”.* (inter 7, para 26–28)

At the same time, some physicians were simply not motivated to continue applying EBM:

*“And well, to say that I’m going to work on it in a structural way, that I schedule it in, no idea when I’m going to find the time, but that’s beside the point, no, I just haven’t got that motivation at the moment”.* (inter 5, para 59)

The need to practice EBM was met primarily by collaborating with other EBM colleagues in case study groups, preferably in case protocol groups, because this approach motivated and activated the participating physicians:

*“If you do it with others, it’s much more efficient because, well, you inspire each other and you think of things that you otherwise wouldn’t have considered. Or just the fact that you agree to do it with someone else, or other people, means you end up doing it earlier than if that wasn’t the case. It’s just like going to the gym – you keep going if you arrange to do it with someone else”.* (inter 3, para 14)

One of the prerequisites mentioned in this context was that the physicians participating in these EBM case study groups enjoyed doing EBM:

*“The main thing is that you have people who enjoy it. Because once you have a couple of people who have fun doing it, it’ll spread. If it becomes a case of feeling forced to do it, then it won’t work at all”.* (inter 3, para 89)

### Facilitating material conditions: access to literature, education in EBM, helpdesk, databases and tools for EBM

Participating physicians stated that an essential condition for being able to work in an evidence-based manner was to have online access to a medical library. Some physicians and offices considered setting up a close collaboration with the regional university, for example via facilities for supporting students of medicine during their internship, as a way to guarantee future library facilities from the university.

*“This access to the library, that’s necessary, and something like that search pyramid tool that the medical library at the academic medical center recently supplied, that can have a lot of added value … searching in an aggregated way”.* (inter 11, para 98)

*“In exchange for (giving education to) the students of medicine from city Q, we could arrange a connection (to the literature) via the University of city Q”.* (inter 10, para 50)

In their efforts to continue working in an evidence-based manner, physicians considered the possibility of support from EBM teachers, through the organization of EBM refresher days as a follow-up to the basic course:

*“The refresher day, I think you’d need to do that at regular intervals. Where you can exchange experiences, perhaps you’ve heard of new developments, and that you can perhaps add some body to it in conjunction with each other”.* (inter 7, para 249)

The facility of an EBM helpdesk was another option proposed as support for evidence-based working:

*“If we’re then looking for information, and we get zero hits for a search query, we could have a helpline to the EBM teachers, who can tell us what we’re doing wrong. Otherwise, you’re just going round in circles”.* (inter 11, para 147)

Additionally, guiding students of medicine to look for answers to questions using EBM was considered as supportive and educational:

*“These students of medicine doing their internship feel more at home in this area, much more at home than we do. Then I would hand over my problem to them. Generally speaking they’re competent. It’s a good system, and fun too. You can learn a lot that way”.* (inter 7, para 46–47)

Many physicians mentioned that, once evidence had been found for a case, results of these EBM efforts should be included in a central database, so that the knowledge gained in this way could be shared and duplication of work efforts would be avoided:

*“Building an anonymized database is a very good idea. It’s not that complicated, if all your EBM findings … if you’ve looked for something, that it can be retrieved later”.* (inter 8, para 636–644)

Google was sometimes mentioned as an alternative, time-efficient resource for acquiring knowledge, but was also questioned as a source for evidence-based working:

*“Of course Google is much more efficient, and your job’s done much quicker. Well, actually I think it’s a pity, when I come to think of it … How efficient is it, in what way? Time-efficient*”. (inter 2, para 145–147)

### Organizational support and demands: time and managerial support; adequate quality assurance policy

Consulting guidelines and other forms of aggregated evidence enabled participating physicians to come up with quick answers: *“… you just look things up and in 15 min you’ve got the answer…”.* (inter 1, para 358) This enhanced the willingness to work in an evidence-based manner, or to continue working that way. However, some recently trained physicians remarked that applying EBM costs time, with the results often being disproportionate to the time and effort spent: *“… I have to get up to all sorts of tricks to find the time, plus the fact that once you’ve spent all that time, the results are unfortunately not always worth it”*. (inter 5, para 6)

Though EBM took time, some physicians wanted to keep up to date on EBM and scheduled time for it: *“… you just have to allocate blocked time for it because otherwise it just won’t happen …”.* (inter 11, para 147) Additionally, for the application of EBM in everyday practice, participating physicians stated it would help if more time was available for reporting evidence-based disability evaluations, in which evidence was sought as underpinning for decision making:

*“That you acknowledge, particularly, that that’s an element we have to pay far more attention and energy to, that’s really important if we want to raise the quality, and that definitely makes a difference, and having the time you need to be able to tell your story effectively”.* (inter 1, para 202–203)

Not surprisingly physicians indicated that to work in an evidence-based way, time must be facilitated in a structural manner, in addition to the physician’s own time:

*“If you want to do it, you need the time for it. And of course it can also be a combination, for me in any case, or for us, it can be a combination of leisure time and work time”.* (inter 11, para 52)

In order to encourage evidence-based working, and also to acquire the resources, such as time and opportunity necessary for this, it was suggested that it was also important to create ‘allies’ among the management and the senior staff physicians.

*“And when I consider lobbying I mean also among the management team, to make clear how important it is, for the client of course, for our own professional sector, that we do have some say in the matter, including towards treating physicians. So that we really are a sparring partner for treating physicians, but also for society as a whole. That you can also now and then support the opinion that someone may perhaps have a complication that can still be treated. And that you can then ensure that action plans are set into motion and that therefore the damage to health can be limited, as well as the resources that will need to be used”.* (inter 6, para 16)

*“Yes, but it’s all about whether the management team is convinced that it (EBM) has added value. If you simply say, well yes, we are able to make better prognoses, manage the dossier stream better, and therefore also save time. And it (better underpinned decisions) probably results in less clients who appeal against the decision, which is also time saved of course. So that could also be an argument for claiming it as work time”.* (inter 1, para 151)

Working in an evidence-based manner could be further encouraged if, for example, the evidence was specified in a separate heading in the disability evaluation report:

*“I find also that you have to use it (evidence) in your reports, which is also now being proposed … Even if you’re only referring to the protocol, that’s evidence too … Just as a standard thing in your reports … You mean just like a standard heading (in the report itself) … information from third parties EBM (proposed subheadings).”* (inter 7, para 129)

Including the degree of substantiation through evidence as a part of the standard quality check was also believed as a stimulus for evidence-based working by the physicians:

*“… quality test (of the report) … Would you include this (evidence) as a chapter there then, or could you take it (evidence) into consideration there … as a controlling function. Indeed, that acts as a stimulus for physicians a little … Yes, and of course you’re not just checking (evidence), you’re also encouraging”.* (inter 10; para 253–257)

## Discussion

In this study we found several motivators and preconditions perceived to promote or hinder implementation of EBM in clinical practice. The participating physicians indicated that EBM improved the quality and attractiveness of their work. They were also clear about what needed to be done to improve the application of EBM in terms of professional competence needed, material conditions facilitated and support and demands from the organization.

### Strengths and limitations

A strength of this study is that we examined the perceptions and experiences of physicians who completed formal education in EBM and who had recent practical experience of its opportunities and problems, as shown in the results. We organized a substantial number of focusgroups (9), including two plenary sessions, and consequently identified many overlapping responses within each theme. However, we hypothesize that we may have included physicans with a somewhat positive attitude towards EBM as we did not include any physicians without recent EBM training who lacked recent motivation or reinforcement. Future studies might therefore consider including physicians whose training in EBM is less recent. In addition, as the participating physicians were interviewed within the EBM course, and the facilitators were acting both as course tutors and researchers, this may have positively skewed any attitudes in favor of EBM. Prior to the start of the course the physicians attitude towards EBM was already highly positive, and remained stable over time [6]. However, we believe the most important concepts and barriers to EBM related to the use of EBM for this group of physicians have been addressed and reported, as similar themes have also been reported in other interview studies within EBM [9, 13–17] among different professionals.

### Comparison with other studies

Similar to other studies among general practitioners [9] and occupational physicians [13, 14], we found that physicians who performed disability assessments believed that EBM enhanced the quality of their practice by keeping up to date with relevant developments, decisions being better underpinned with evidence and clients ultimately benefitting from the application of EBM. Unlike Shuval et al. [9] and Hugenholtz et al. [13], we found that physicians perceived that the services of their organization could be improved as a consequence of using evidence, another aspect of quality of their practice. In our study consultation with medical colleagues and job satisfaction also improved [13].

When physicians are able to raise the level of medical content of their profession by using the medical literature, it could enhance their job satisfaction [13]. Some physicians perceived themselves as not sufficiently skilled or motivated for EBM practice. Considering the mean increase in self-efficacy in our RCT, and even a consequent change in behavior, this finding deserves attention. Likely a subgroup felt less confident. Hugenholtz et al. made a similar observation, despite the overall favorable results including enhanced evidence-based advice after training [13]. These authors found that peer group sessions facilitated the sharing of knowledge, a concept that is reflected in our subthemes of ‘more appreciation and job satisfaction’ and ‘collaboration’, in which knowledge exchange is thought to facilitate evidence-based practice. Likewise, Shuval et al. mention the need to meet regularly with colleagues experienced in information retrieval and to jointly look for answers to medical questions [9].

In line with several studies [2, 9, 13, 15], physicians in our study perceived lack of time as a barrier for applying EBM. Recently, a study in this journal about perceptions of Norwegian doctors towards EBM confirmed that both the organization and the physicians themselves should invest [16] in EBM, similar to our finding. A novel aspect in our view is the finding that physicians often regarded the output of their EBM efforts as being disproportionate to the time invested. This may have to do with a lack of experience in information retrieval as our physicians recently completed their course. In another study general practitioners achieved an efficient trade-off between maximizing search success and information reliability, and minimizing search time [17]. Another explanation may be lack of recognition for the use of EBM in daily practice and not receiving a reward from management or colleagues for these extra efforts. As with most educational interventions, keeping up with EBM is more difficult over time, unless skills and knowledge are refreshed. This is also why in educational interventions refresher courses are advocated to stimulate the continued use of EBM.

### Implications for practice and further research

In order to further increase competency and motivation participating physicians expressed the wish to receive EBM refresher courses and for a helpdesk for EBM questions, a proposal also mentioned by Shuval et al. [9]. As a result of the first request, EBM refresher courses have already started and provide input for the further refinement of our EBM program towards enhancing evidence-based disability assessment in the Netherlands. The possibilities of a helpdesk or consulting team to facilitate initiatives from the professionals themselves, as requested, is still under consideration within the Dutch National Institute for Employee Benefit Schemes (UWV). Similar facilities set up for general practitioners in the United Kingdom and the Netherlands have been evaluated positively [18, 19].

Involving more medical internship students who, in general, are better skilled in EBM can help physicians learn from EBM assignments done by these interns. The expressed need, also in other disciplines [9, 13], for collaboration with other colleagues to practice EBM, should be embraced in order to further implement EBM in the field of disability evaluations. A number of groups already started after the course. Evidence-based practice could be facilitated if the organization, manager and medical colleagues all see the application of EBM as a priority.

Conversely, the increased expertise of these physicians could also help the organization in their efforts in improving products and collaboration with other organizations. This contributes directly and explicitly to the ambition of the Dutch National Institute for Employee Benefit Schemes to become the leading expert organization in disability evaluation in the Netherlands, competing with the best institutes in other countries. Needless to say, a good knowledge infrastructure – and its maintenance – with online access to requested evidence, is an absolute prerequisite [20].

Future research could include the evaluation of the introduction of new EBM tools, such as online access to up-to-date search strategies and search engines, EBM case-based tutorials, online pre-appraised literature or even an EBM question repository, to facilitate evidence-based work. Furthermore, investigating the foraging approach that aims at an efficient trade-off between maximizing search success and information reliability, and minimizing search time [17] could enable physicians evaluating disability to work more evidence based.

## Conclusion

Physicians are inclined to use EBM because they perceive it as improving the quality of their work and as a factor making their work more attractive. In addition to personal investments and gains by physicians performing disability evaluations, organizations that employ these physicians should further facilitate the uptake of evidence in practice.

### Ethical approval

In this cluster RCT approval was sought from the research ethics committee of the Academic Medical Center, University of Amsterdam. However, as this constituted the evaluation of an educational intervention with physicians, written exemption of ethics approval was received from the committee secretary.

## Appendix

### Assignment for physicians prior to group discussion: creating a personal action plan

How can I keep using my EBM skills?

In order to proceed with evidence-based practice we would like to encourage you to make a personal action plan. The questions *“How would I like to see myself as a practicing EBM physician in six months’ time?”* and *“What do I need to realize this?”* are central in this.

Answer these questions in your action plan for the upcoming six months, approximately one page and a half. Do this based on the following pattern: purpose, resources, actions. See the next page. Fill in this form electronically.

On behalf of all EBM teachers and tutors: thank you and good luck with your plan! Mail this plan to RK a week before the start of the course, at the latest.

Please bring a printout of your plan and three extra copies for the discussion on course day 5.

Personal action plan

Name: ……………………………………………………..

In formulating this personal action plan, two questions should be kept in mind:

- How would I like to see myself as a practicing EBM physician in six months time?
- What do I need to realize this vision?

1. Formulate your personal goals.
2. Which resources (instruments) will you use?
3. What are you actually going to do? What are your personal actions?

Describe terms of: When? Why? How often? Where? In which situation? i.e. measurable.

Use as much space as you need.

## References

[1] Dawes M, Summerskill W, Glasziou P, Cartabellotta A, Martin J, Hopayian K (2005). Sicily statement on evidence-based practice; second international conference of evidence- based health care teachers and developers. BMC Med Educ.

[2] Coomarasamy A, Khan KS (2004). What is the evidence that postgraduate teaching in evidence-based medicine changes anything? A systematic review. BMJ (Cli Res Ed).

[3] Forsetlund L, Bjorndal A, Jamtveldt G, O'Brien MA, Wolf F, Davis D (2009). Continuing education meetings and workshops: effects on professional practice and health care outcomes. Cochrane Database Syst Rev.

[4] Marinopoulos SS, Dorman T, Ratanawongsa N, Wilson LM, Ashar BH, Magaziner JL (2007). Effectiveness of continuing medical education. Evid Rep Technol Assess.

[5] Davis D, Davis N (2010). Selecting educational interventions for knowledge translation. CMAJ.

[6] Kok R, Hoving JL, Smits PB, Ketelaar SM, van Dijk FJ, Verbeek JH (2013). A clinically integrated post-graduate training programme in evidence-based medicine versus 'no intervention' for improving disability evaluations: a cluster randomised clinical trial. PLoS One.

[7] Kok R, Hoving JL, Verbeek JH, Schaafsma FG, van Dijk FJ (2011). Integrating evidence in disability evaluation by social insurance physicians. Scand J Work Environ Health.

[8] Kok R, Hoving JL, Verbeek JH, Schaafsma FG, Smits PB, van Dijk FJ (2008). Evaluation of a workshop on evidence-based medicine for social insurance physicians. Occup Med (Lond).

[9] Shuval K, Shachak A, Linn S, Brezis M, Feder-Bubis P, Reis S (2007). The impact of an evidence-based medicine educational intervention on primary care physicians: a qualitative study. J Gen Intern Med.

[10] Strauss AL, Corbin J (1990). Basics of qualitative research: Grounded theory procedures and techniques.

[11] Tong A, Sainsbury P, Craig J (2007). Consolidated criteria for reporting qualitative research (COREQ): a 32-item checklist for interviews and focus groups. Int J Qual Health Care.

[12] Donabedian A (1966). Evaluating the quality of medical care. Milbank Mem Fund Q.

[13] Hugenholtz NI, Schaafsma FG, Schreinemakers JF, van Dijk FJ, Nieuwenhuijsen K (2008). Occupational physicians’ perceived value of evidence-based medicine intervention in enhancing their professional performance. Scand J Work Environ Health.

[14] Schaafsma FG, Hugenholtz N, de Boer A, Smits PA, Hulshof C, van Dijk FJH (2007). Enhancing evidence-based advice of occupational health physicians. Scand J Work Environ Health.

[15] Grol R, Grimshaw J (2003). From best evidence to best practice: effective implementation of change in patients' care. Lancet.

[16] Ulvenes LV, Aasland O, Nylenna M, Kristiansen IS (2009). Norwegian physicians' knowledge of and opinions about evidence-based medicine: cross-sectional study. PLoS One.

[17] Dwairy M, Dowell AC, Stahl JC (2011). The application of foraging theory to the information searching behaviour of general practitioners. BMC Fam Pract.

[18] Swinglehurst DA, Pierce M, Fuller JC (2001). A clinical informaticist to support primary care decision making. Qual Health Care.

[19] Verhoeven AA, Schuling J (2004). Effect of an evidence-based answering service on GPs and their patients: a pilot study. Health Info Libr J.

[20] van Dijk FJ, Verbeek JH, Hoving JL, Hulshof CT (2010). A knowledge infrastructure for occupational safety and health. J Occup Environ Med.

